# Can Eye Tracking Help Assess the State of Consciousness in Non-Verbal Brain Injury Patients?

**DOI:** 10.3390/jcm13206227

**Published:** 2024-10-18

**Authors:** Grzegorz Zurek, Marek Binder, Bartosz Kunka, Robert Kosikowski, Małgorzata Rodzeń, Danuta Karaś, Gabriela Mucha, Roman Olejniczak, Agata Gorączko, Katarzyna Kujawa, Anna Stachowicz, Karolina Kryś-Noszczyk, Joanna Dryjska, Marcin Dryjski, Jarosław Szczygieł

**Affiliations:** 1 Department of Biostructure, Wroclaw University of Health and Sport Sciences, 51-612 Wrocław, Poland; 2 Institute of Psychology, Jagiellonian University, 30-060 Krakow, Poland; marek.binder@uj.edu.pl; 3 Research & Development Department, AssisTech, 80-180 Gdansk, Poland; bartosz.kunka@assistech.eu (B.K.); robert.kosikowski@assistech.eu (R.K.); 4 Polskie Centrum Rehabilitacji Funkcjonalnej VOTUM, 30-723 Krakow, Poland; malgorzata.rodzen@pcrf.pl (M.R.); danuta.karas@pcrf.pl (D.K.);; 5 Neurorehabilitation Clinic, 54-530 Wroclaw, Poland; roman.olejniczak@gmail.com (R.O.); agagoraczko@gmail.com (A.G.); katarzyna.kujawa@awf.wroc.pl (K.K.); annimast@gmail.com (A.S.); 6 Centrum Opieki i Rehabilitacji, 42-200 Czestochowa, Poland; karolinakrysum@gmail.com (K.K.-N.); jcogiel@op.pl (J.D.); marcin.dryjski@gmail.com (M.D.); 7 Szpital Powiatowy, 42-400 Zawiercie, Poland; jwszczygiel@gmail.com

**Keywords:** eye tracking, disorders of consciousness, assessment, severe brain injury

## Abstract

**Background/Objectives:** Developments in eye-tracking technology are opening up new possibilities for diagnosing patients in a state of minimal consciousness because they can provide information on visual behavior, and the movements of the eyeballs are correlated with the patients’ level of consciousness. The purpose of this study was to provide validation of a tool, based on eye tracking by comparing the results obtained with the assessment obtained using the Coma Recovery Scale-Revised (CRS-R). **Methods:** The mul-ti-center clinical trial was conducted in Poland in 2022–2023. The results of 46 patients who were not able to communicate verbally due to severe brain injury were analyzed in this study. The state of consciousness of patients was assessed using the Minimally Conscious State Detection test (MCSD), installed on an eye tracker and compared to CRS-R. The examinations consisted of performing the MCSD test on patients five times (T1–T5) within 14 days. Collected data were processed based on the FDA and GCP’s regulatory requirements. Depending on the nature of the data, the mean and standard deviation, median and lower and upper quartiles, and maximum and minimum values were calculated. Passing–Bablok regression analysis was used to assess the measurement equiva-lence of the methods used. **Results:** There was no difference between the MCSD and CRS-R in the raw change between T5 and T1 time points, as well as in the total % of points from all time points. The MCSD results from each time point show that at least the first two measurements serve to famil-iarize and adapt the patient to the measurement process, and the third and next measurement should be considered reliable. **Conclusions:** The results indicated a significant relationship be-tween the scores obtained with MCSD and CRS-R. The results suggest that it seems reasonable to introduce an assessment of the patient’s state of consciousness based on eye-tracking technology. The use of modern technology to assess a patient’s state of consciousness opens up the opportunity for greater objectivity, as well as a reduction in the workload of qualified personnel.

## 1. Introduction

The diagnosis of brain injuries and their consequences in people with impaired consciousness is a complex process that requires a diverse approach and the use of a variety of diagnostic methods. Its main goal is to determine the cause of the disorder of consciousness as well as to identify possible brain damage that may be its consequence. The final diagnosis often requires interdisciplinary cooperation between neurologists, neurosurgeons, intensive care specialists, and other medical disciplines [1].

The primary diagnostic tool is a neurological examination, which includes the evaluation of brain functions such as pupillary responses, reflexes, eye movements, and responses to external stimuli [2]. Other techniques such as CT, MRI [3], fMRI [4], and PET [5,6] are also used. These methods allow accurate imaging of brain structures and detection of possible damage [7]. In addition, to monitor a patient’s vital signs, for example, blood pressure, saturation levels as well as electrical activity of the brain in an EEG study are assessed [8]. These data can provide valuable information about brain activity and possible abnormalities but serve primarily to make a proper diagnosis to determine the correct clinical management of a patient in a state of disordered consciousness.

The classical approach to assessing the state of consciousness of patients with severe brain damage in Poland involves the Glasgow Coma Scale (GCS) as the main tool used. Experience shows that this tool, which is widely known in clinical practice, has both advantages and undeniable disadvantages. The main advantages of the GCS include its universality of use, as well as its speed and simplicity of execution, which are important, especially in neurological emergencies [9], the standardization and comparability of results between different patients, and the monitoring of changes over time [10]; some studies suggest that the GCS may be useful for predicting the prognosis of patients after head trauma, which has important clinical implications for planning care and rehabilitation [11]. In addition to the aforementioned advantages, the GCS has undeniable disadvantages, which include limited sensitivity in detecting changes in the state of consciousness, especially in patients with mild traumatic brain injury or reduced responsiveness [9], lack of assessment of some important neurological functions (eye movements or communication ability), as the GCS mainly focuses on assessing motor and verbal responses to the exclusion of other important neurological functions [12], and subjectivity of the assessment as interpretation of the results may depend on the experience of the assessor [13].

Regardless of the stated facts, Teasdale et al. (2014), writing about the GCS, point out the need to consider other aspects of neurological assessment [14].

Recently, in the face of increasing criticism of the GCS, another, more diagnostic, and more precise tool for assessing the state of consciousness of patients has begun to be used, which is the Coma Recovery Scale-Revised (CRS-R) [15]. It is a diagnostic scale for assessing the state of consciousness in people with disorders of consciousness (DOCs). It is more complex than the GCS, as it considers a wider range of neurological functions and responses (including sensory responses, eye movements, verbal responses, and limb movements) to assess the patient’s condition. The main advantages of the CRS-R include its ability to assess the state of consciousness more accurately than other abbreviated/simpler scales such as the GCS [16], and its sensitivity, allowing it to distinguish between a state of minimal consciousness (MCS) and a state of unresponsive wakefulness (UWS), still referred to as a vegetative state in some countries; this is crucial for treatment, prognosis, and patient care planning [16]. In addition, it has the advantage of standardizing the assessment, which allows for a reproducible and reliable evaluation of patients’ state of consciousness; this is particularly important given the monitoring of rehabilitation progress and treatment evaluation [17]. In addition, according to Seel et al. 2010 [17] and Stender et al. 2014 [18], it may be more sensitive to subtle changes in state of consciousness than other scales, making it possible to better monitor patient responses to treatment and rehabilitation. However, it is worth noting the criticisms leveled against it. A rather important limitation is that it requires training and experience, as it is a more complex scale than the GCS. Thus, specialized training and staff experience in its use are required to ensure reliable and consistent results [19]. Due to its complexity compared to the GCS, the CRS-R is also much more time-consuming and for this reason, may not be used in emergency cases [18]. Critics of the method also point out that some components of the CRS-R assessment (e.g., assessment of sensory responses or eye movements) can be prone to subjective interpretations, which can affect the consistency of results [20].

Other less common tools for assessing a patient’s state of consciousness are (1) sensory response assessment (e.g., pain, touch, sound), which analyzes stimuli to which the patient responds through changes in physiological parameters, e.g., changes in blood pressure and autonomic responses [21,22]; (2) brain imaging studies such as CT or MRI, which are used to assess brain structure and identify possible damage; they can help determine the cause of a patient’s condition and predict further rehabilitation [23]; (3) neurophysiological studies, such as EEG or evoked potential studies, which provide information on the electrical activity of the brain in patients who do not communicate verbally. These methods can be useful in assessing the degree of brain activity and nervous system function [24,25,26,27].

In general, it can be concluded that assessing the state of consciousness of patients who do not communicate verbally requires a variety of methods that can take into account motor and sensory responses, as well as imaging and neurophysiological studies. In light of the information presented above, despite some criticisms, it is believed that the CRS-R is one of the key and most objective tools today for assessing the level of consciousness in these patients, but it is also important to use other diagnostic techniques to obtain a comprehensive clinical picture [17]. Tailoring the diagnostic approach to the individual patient’s needs and capabilities is crucial for effective case management of patients with disorders of consciousness. Various aspects of patient assessment are important and the final diagnosis should result from a clinical consensus approach and standardized neurobehavioral assessment of patients in a state of minimal consciousness [28,29].

However, significant developments in modern technology, particularly eye-tracking technology, are opening up new possibilities for diagnosing patients in a state of minimal consciousness. An excellent tool for such an assessment is the eye tracker, whose capabilities can be used to detect eye movements. This technology can provide information on visual behavior, such as fixations and saccades [29,30,31]. The way the eyeballs move and the precision of their movements are correlated with the patients’ level of consciousness, making it possible to identify patterns of eye responses characteristic of different states, such as a state of minimal consciousness [32]. Therefore, in determining a patient’s state of consciousness, assessment of responses to visual stimuli can/should be taken into account to correctly identify eye responses in response to external stimuli [33]. Of course, the use of eye trackers is associated with certain limitations. These include the complexity of interpreting the results, as the information obtained can be complicated and requires specialized knowledge to accurately understand the meaning of eye movements in the context of assessing the state of consciousness [34]. In addition, there are individual differences in eye movement patterns, which can make it challenging to identify unambiguous indicators of state of consciousness based on eye tracker data analysis [35]. Regardless of the conditions presented, it seems that the use of modern ophthalmographic technology, making the assessment of the patient’s state of consciousness independent of the individual competence of the examiner, and therefore eliminating the factor of subjectivity in this key assessment, represents the future in the diagnosis of neurological disorders and should be developed. At the same time, further research and development of eye-tracking technology can contribute to a better understanding and use of this tool in the diagnosis of patients with disorders of consciousness. Therefore, the purpose of this study was to develop a novel scale for assessing a patient’s state of consciousness using eye-tracking technology and to provide initial validation of the tool through initial validation of the C-EYE X tool based on comparing the results obtained with the scores obtained using the CRS-R.

## 2. Materials and Methods

### 2.1. Participants and Study Design

The study was approved by the Senate Committee on Research Ethics at the Wrocław University of Health and Sport Sciences, Poland (decision no. 11/2022). All activities were conducted following ethical standards for responsible human research, as well as taking into account the principles of the 1975 Declaration of Helsinki. Data and methods of analysis are available to qualified researchers upon request.

### 2.2. Study Participants

The multi-center clinical trial was conducted during the period from July 2022 to February 2023 at five inpatient care centers in Poland with patients undergoing neurorehabilitation due to a history of severe brain injury (Krakow, Sawice, Czestochowa Wroclaw, and Zawiercie). Inclusion in the study was based on medical qualification, after the patient met the inclusion criteria, i.e., (1) age ≥ 18 years, (2) legal guardian’s consent to participate in the study and access to medical records, (3) a medical diagnosis indicating damage to the central nervous system (CNS), (4) possession of and access to imaging examination reports (MR or CT) and, as an alternative, oculometric tests, ophthalmic examination, and hearing assessment, (5) providing a list of medications used by the patient that may affect the results obtained in tests of cognitive function, (6) physician’s approval (e.g., neurologist, neurosurgeon, internist) for participation in the clinical trial, following the review of the study protocol, including the ability to communicate by sight only (no verbal, sign language, or other communication possible), the absence of dementia and aphasic disorders prior to the event that caused the CNS damage and the patient’s current condition, and preservation of at least one functioning eyeball (ability to establish cooperation with an eye tracker).

The exclusion criteria for patient participation in the study were (1) a visual defect (refractive defect) diagnosed before the injury, requiring the use of glasses with lenses of more than ±3 diopters, and (2) the inclusion of pharmacological treatment during the study (observation), which may affect the patient’s cognitive functioning—both in terms of an increase in cognitive abilities, as well as their impairment/dementia.

**Figure 1:**
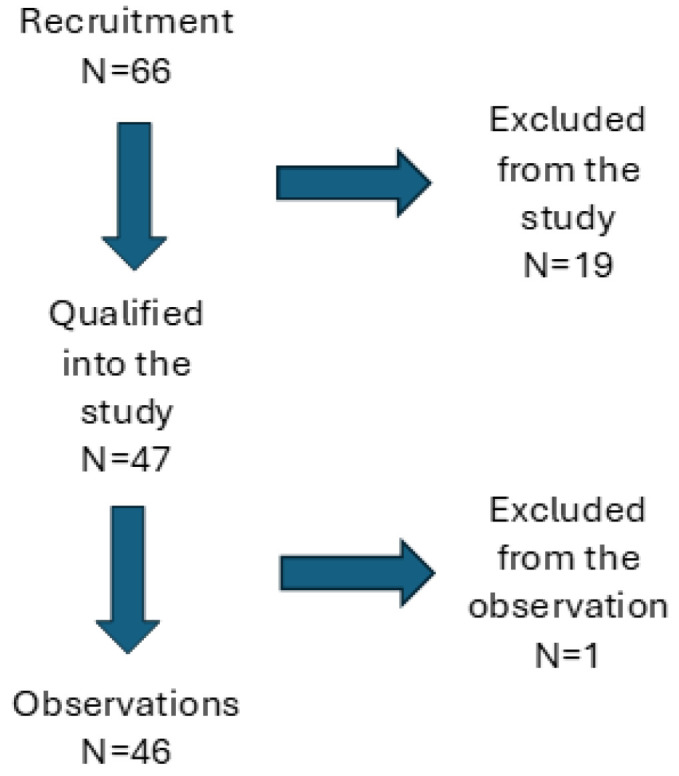
Flow chart of study enrolment, allocation, and analysis.

A total of 66 patients were recruited for the study, but for various reasons (failure to meet the inclusion criteria, failure to perform the CRS-R test during the study, as well as ill health and death of 1 subject), the results of 46 patients are analyzed in this study (Figure 1). Men predominate among them (N = 27, i.e., 58.7%), and the number of women is N = 19 (48.4%).

The etiology of brain damage in the patients whose results were analyzed varied; the most common cause was craniocerebral trauma (34%) and stroke (32%). Details of etiology and demographics are shown in Table 1.

### 2.3. Study Procedure and Data Collection Methods

The state of consciousness of patients qualified for the study was assessed using the Minimally Conscious State Detection test (MCSD), installed on the C-EYE X device. This test, which includes assessments of auditory function, visual function, visual–auditory integration, following commands, orientation, and pain localization, was created by a team of neuropsychology and medical specialists (physicians, nurses, physiotherapists) in collaboration with computer scientists. The team’s task was to prepare a test diagnosing auditory and visual functions and adapt it to an electronic version based on eye-tracking technology for patients who do not communicate verbally.

The data used in the study were collected using an eye-tracking device known as the C-Eye X, which has the following specifications: sampling rate of 33 Hz, accuracy of 0.5 degrees of visual angle, speed threshold of 40 cm/s. The C-Eye device has a 19-inch screen that is mounted on a special mobile tripod, which can be rotated to place the device’s screen in front of the subject’s face at a distance of 60 cm. At the bottom of the monitor is an eye tracker that emits infrared (IR) radiation, which does not affect the patient’s work with the device. Depending on whether the patient has one or both eyeballs functional, different eye tracking modes (monocular or binocular) can be used. Prior to the test, a single-point calibration was conducted on each participant to determine the location of the patient’s eye fixation point (the 2D image from the IR camera is processed on the device’s screen), with the position of the eyeball estimated by the eye tracker based on the position of the center of the pupil and two characteristic infrared reflections on the cornea of the eye. During calibration, the patient was asked by the examiner to look at and hold his or her gaze on a red flashing dot with a white border, displayed in the center of the screen. Analysis of the patient’s gaze fixation continued until the system correctly detected the position of the eyeballs. If the patient was unable to fixate their eyeballs, the system informed them after 10 s that the calibration had failed and the task could not be continued. The eye-tracking system used can compensate for small head movements, so there was no need for patients to stabilize their heads.

The Minimally Conscious State Detection (MCSD) diagnostic tool consists of a series of clinical trials intended to measure meaningful eye movement responses that are diagnostic for the detection of consciousness. The test trials are grouped into six subscales (the names of included trials are given in parentheses): auditory function (Auditory Startle Reflex), visual function (Visual Fixation, Visual Pursuit, Letter Recognition), audio-visual integration (Recognition of Sound Stimulus—with color and black-and-white visual stimuli), command following (Object Selection, Screen Area Selection), autopsychic orientation (Autobiographical Questions—own name, birth year), and localization to noxious stimulation (Response to Pressure). In all trials, test scoring is based on detecting eye movements to target stimuli or parts of the screen, with two exceptions. Trials included in auditory function (Auditory Startle Reflex, assessing the integrity of the lower parts of the auditory pathway) and localization to noxious stimulation (Response to pressure applied to fingers or toes, measuring cortically mediated response to noxious stimulation) are based on observing the motor behavior of the patient.

The visual stimuli displays were always divided into two halves representing two target areas. The default setup was the horizontal arrangement of the target areas. When only vertical eye movements were observed, the arrangement of target areas was changed to a vertical one.

In each trial, the patient’s response was scored if the dwell time on the target area exceeded 1.2 s. In most trials, the response window duration was 1 min.

Prior to the actual study (visits 2–6), as part of the initiating visit (visit 1), socio-demographic data were collected about the patients, and an interview was conducted to establish a diagnosis of the patient’s condition; information was also collected on the Glasgow Coma Scale (GCS) scores; the average score of the study participants was 7 (SD = 1.7). Basic information about the patients is included in Table 2.

The Glasgow Coma Scale (GCS) was used to assess the level of consciousness in patients. The GCS evaluates basic behaviors related to eye opening and verbal and motor responses. The minimal score is 3 and the maximal 15, with the higher scores indicating less severe impairment of consciousness [9]. Although the GCS is better suited to assess the neurological status in acute-stage patients, the GCS is widely used in Poland and also in the clinical diagnosis of chronic DOC patients.

The proper examinations (visits no. 2–6, further indicated as T1, T2, T3, T4, and T5 measurement points) consisted of performing the MCSD test five times within 14 days, with successive examinations according to the protocol to be separated by at least a 1-day interval. The examinations were performed by an experienced neurorehabilitation specialist, whose competence was confirmed by a certificate qualifying them to work with the eye tracker, as well as trained in working with the CRS-R. The duration of one examiner’s visit to a patient was variable, ranging from 40 to 60 min, and depended on the degree of cooperation between the patient and the therapist performing the MCSD test. Each of the five MCSD test examinations was preceded by the administration of the CRS-R.

The Polish version of the CRS-R was used to assess the neurocognitive status of the patients [36]. The scale consists of 23 items covering basic perceptual, motor, and cognitive abilities. The items are grouped into six subscales: auditory, visual, motor, and oromotor/verbal, as well as communication abilities and arousal. The CRS-R is regarded as the most widely used scale for the assessment of chronic DOC patients, providing differential diagnosis, prognostic assessment, and treatment planning [15].

The last visit (no. 7) was dedicated to reassessing the patient’s condition and was conducted by a specialist.

To facilitate data collection and integration, all patient data were processed using the GoInsights™ platform (https://www.goinsights.net/). It is based on the FDA’s 21 CFR Part 11 and GCP 5.5.3 regulatory requirements for electronic data. The nature of GoInsights™’s security features ensures a high level of data quality during the data recording process, as it allows only the data provided for in the specifications to be entered, as well as minimizes the risk of incomplete data entry. All data were recorded in the electronic patient record (eCRF).

### 2.4. Data Analysis

The investigation of the results of this study was preceded by the preparation of a Statistical Analysis Plan, which was then used to conduct the specific statistical analyses. Depending on the nature of the data, the mean and standard deviation, median and lower and upper quartiles, and maximum and minimum values were calculated. The distribution of variables was assessed using a quantile–quantile (Q-Q) plot and the results of the Shapiro–Wilk test. The homogeneity of variance was assessed using Levene’s test. A statistical significance level of α < 0.05 was adopted.

Raw scores in CRS-R and C-EYE X MCSD were normalized to the percentage of the maximum possible total score for each tool (indicated as Total CRS-R % score and Total C-EYE X MCSD % score, respectively).

For each subsequent visit proper (visits 2–6), the percentage changes in total and subscale scores compared to visit 2 are presented. Passing–Bablok regression analysis was used to assess the measurement equivalence of the methods used. All analyses were performed using the R statistical package, ver. 3.6.3 [37].

## 3. Results

A comparison of the MCSD and CRS-R results is presented based on the calculated percentages of the maximum test score (Table 3). The mean values of the total percentage scores obtained in the MCSD are lower compared to values calculated for the CRS-R scores for each visit.

There was no difference between the MCSD and CRS-R in the raw change between the T5 and T1 time points (3.84% ± 16.95% vs. 3.00% ± 10.96%; *p* = 0.78), as well as in the total % of points from all time points (39.56% ± 20.35% vs. 47.90% ± 27.04%; *p* = 0.10) (Table 3).

The interquartile range (Q1–Q3) of total % points on the MCSD was lower compared to the interquartile range of total % points on the CRS-R for each visit. The coefficients of variation are similar for both tests. For the CRS-R, the upper quartile values at each time point were higher than for the MCSD. This means that the top 25% of patients achieved values (at the five time points) no lower than the range of 65.22–73.91% in the CRS-R, and no lower than the range of 36.36–54.55% in the MCSD.

The MCSD results from each time point show that at least the first two measurements serve to familiarize and adapt the patient to the measurement process, and the third measurement should be considered reliable. There are significant differences in mean values between time points T1 vs. T2 vs. T3.

The results in Table 4 indicate that there was no significant difference between the two tests, regardless of time (*p* = 0.10), but there was a statistically significant effect of time (*p* < 0.001) and interaction between time point and test type (*p* < 0.01). There are significant differences in mean percentages between different time points (T1 vs. T2 vs. T3) for the MCSD (*p* < 0.001), but not for the CRS-R (*p* = 0.35).

For the data obtained, statistically significant and moderate correlations were found between the C-Eye test and CRS-R at each time point (Table 5).

**Figure 2:**
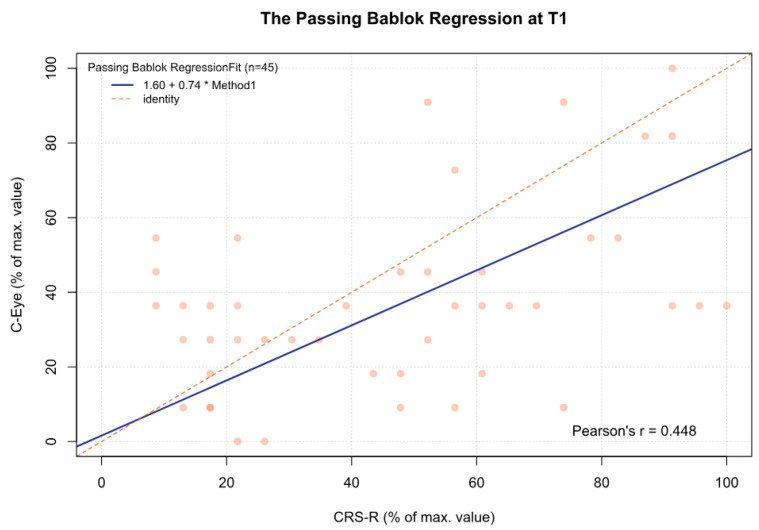
The Passing–Bablok regression between the MCSD and the CRS-R at the T1 time point; pcorr < 0.01.

**Figure 3:**
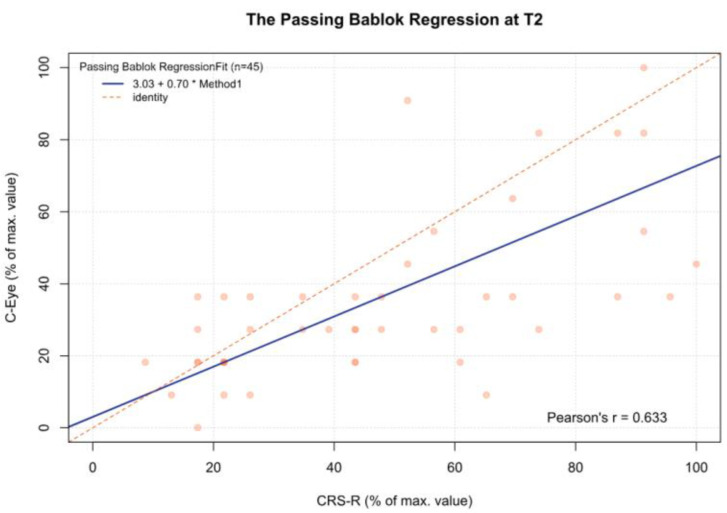
The Passing–Bablok regression between the MCSD and the CRS-R at the T2 time point; pcorr < 0.01.

**Figure 4:**
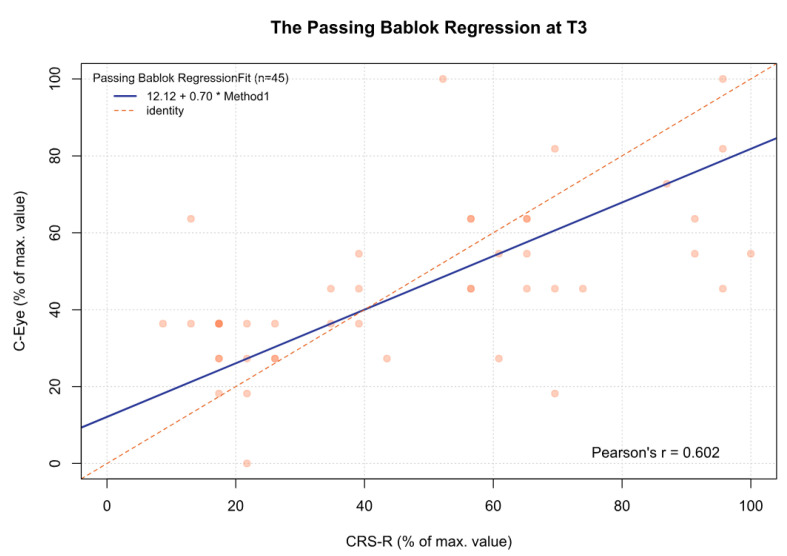
The Passing–Bablok regression between the MCSD and the CRS-R at the T3 time point; pcorr < 0.01.

**Figure 5:**
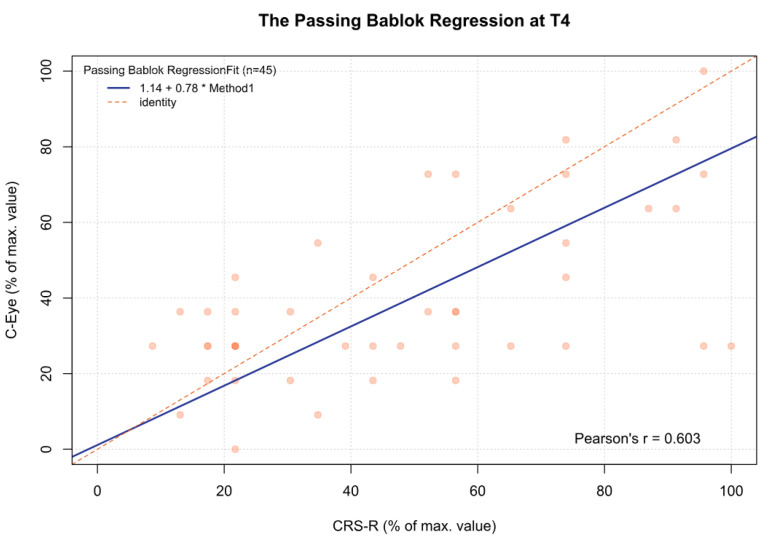
The Passing–Bablok regression between the MCSD and the CRS-R at the T4 time point; pcorr < 0.01.

**Figure 6:**
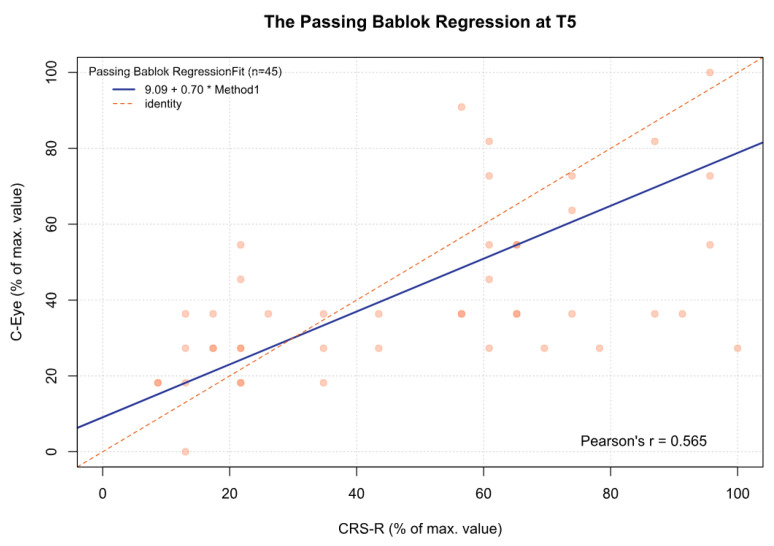
The Passing–Bablok regression between the MCSD and the CRS-R at the T5 time point; pcorr < 0.01.

**Figure 7:**
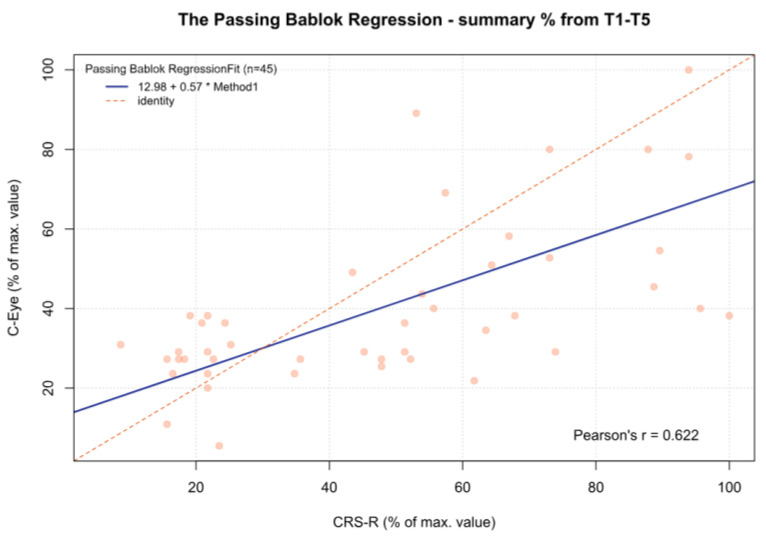
The Passing–Bablok regression between the MCSD and the CRS-R at the T5 time point; pcorr < 0.001.

For the MCSD, significant differences were observed between time point T3 and time points T1 (*p* < 0.05), T2 (*p* < 0.001), and T4 (*p* < 0.05). Other comparisons were statistically insignificant. In addition, for time point T5 and the sum from T1 to T5, there was a significant deviation between both tests, which increased as the CRS-R value increased (β1 = 0.70 and β1 = 0.57, respectively) (Figure 2, Figure 3, Figure 4, Figure 5, Figure 6 and Figure 7, Table 6). This again means that patients scoring higher on the CRS-R at T5 and the sum of all time points have progressively lower values on the MCSD.

## 4. Discussion

The challenges of modern emergency medicine, one of the important criteria of which is the speed of establishing the correct diagnosis and making decisions, are met by the dynamic development of new technologies [38,39,40]. They are increasingly being used in various clinical areas and provide serious support, often replacing previously used methods. In making a proper diagnosis, special attention is paid to its accuracy, as it is the basis for further therapeutic management [41,42]. One of the factors that affect the accuracy of the decisions made is a sufficiently high level of staff training so that the diagnoses made are comparable and as independent as possible of the subjective assessment of the examiner [43]. However, given the emerging misdiagnoses in patient assessment, which are sometimes very common in patients with severe brain damage, it is natural to look for new solutions that make the patient’s clinical assessment independent of the/subjective judgment of the examiner [44]. This is indicated by the limitations of the GCS and CRS-R demonstrated in earlier studies [12,13,18,20]. The results of assessing patients’ state of consciousness, obtained with a novel MCSD tool using eye movement tracking technology, were compared with another scale with the same purpose but different diagnostic sensitivity, the CRS-R.

The average results of the individual measurements (T1–T5) of the patients’ state of consciousness made with the CRS-R are quite similar and not statistically significant from the first to the last examination, which seems to be an advantage of the method used. It should be taken into account, however, that this may be due to a similar—investigator-dependent—way of assessing the patient in subsequent trials (T2–T5), or the investigator’s suggestion of the assessment obtained in the first examination of a given patient. This line of reasoning seems to be confirmed by the results recorded with the MCSD, whose variability is greater from one measurement point to another, and in the extreme case (T2 vs. T3 comparison), the results differ by more than 11% (34.55% vs. 45.86%). The time factor is therefore important for repeated measurements obtained with the eye tracker. In our study, it seems that the first two measurements of the patients’ state of consciousness recorded with the MCSD are more for familiarization with the device, and the third measurement is the highest, while the next two measurements are similar. Previous in-house experience indicates that such even significant variability in cognitive test results or state of consciousness is quite common, and for some cognitive functions can range from complete lack of cooperation (inability to perform tasks) to full cooperation with the therapist (all tasks performed correctly) [45]. It would therefore be reasonable to assume that the therapist’s ability to cooperate with the patient, and therefore to obtain certain results in cognitive tests, may show significant fluctuation between studies. In this context, the results of a test using MCSD, i.e., a mode of testing that is assumed to make the obtained results independent of the examiner, seem to be more useful and at the same time may correspond more closely to the patient’s actual condition. For this reason, repeated measurement with the MCSD tool is also justified, as due to the interfering factors of the patient’s lack of preparation and the variability of the results obtained by the patient, a single measurement may be unreliable/unreliable.

How to use both tools (CRS-R and MCSD) to clinically assess a patient’s state of consciousness remains an important issue. The duration of a single test with each tool is similar, ranging from 45 to 60 min. In a situation where, in successive measurements, the score obtained in the MCSD test is lower than that obtained in the CRS-R with a statistically significant correlation between the two, it can be assumed that the assessment of the patient’s state of consciousness using the eye tracker appears to be more “conservative”. However, it should be emphasized that due to the complete novelty of our study, the range of MCSD results is not empirically known; another thing is that this may also be related to the nature of the study group. Given that the MCSD assessment is based largely on the assessment made by the technological tool, and not on the assessment made by the researcher/therapist, it can be said that its use gives security to the assessment and could be a serious argument for taking further therapeutic steps, including the use of other assessment methods. It could therefore be used as a screening tool in assessing the state of consciousness of patients with severe brain damage. This meets societal expectations, one of the goals of which is the greater incorporation of a variety of methods for assessing a patient’s clinical condition to more objectively evaluate the patient’s state of health [45,46].

The difference in results between the CRS-R and MCSD may also be related to the different nature of the patient’s participation in assessing his state of consciousness. In the case of the MCSD test, a high degree of patient responsiveness is required, due to the need for their cooperation with the therapist through the use of eye movements. Each of the tests that make up the final state-of-consciousness score requires such patient interaction; hence—with increasing fatigue of the muscles that control the eyeball movements—the final results may depend on the patient’s previous experience with the eye tracker. Studies indicate that oculomotor training conducted as part of neurorehabilitation with an eye tracker improves the efficiency of the eye muscles and increases their control so that the time in which the patient can work with the eye tracker increases [47]. This is because the muscles controlling eye movements are striated, and are therefore subject to conscious control, like other such muscles. The fact that several patients with UWS diagnosis as obtained with CRS-R were nonetheless able to take part in the diagnostic test requiring directed eye movements, highlights the potential advantage of the eye tracker method as more sensitive for detecting such movements, and at the same time perhaps reveals limitations of techniques for detecting fixation and visual pursuit that are used in CRS-R. This may mean that the inclusion of eye trackers in the neurorehabilitation process may ultimately affect the state-of-consciousness results obtained in tests based on eye-tracking technology. This issue definitely needs further experimental scrutiny to identify both patient-related and tool-related factors that might be responsible for this phenomenon.

A separate issue, which may be important in the process of supporting a patient’s neurorehabilitation, remains the fact that the patient is involved in the evaluation of the state of consciousness. In a situation in which they can influence this assessment, through their conscious involvement, they move more and more out of the role of an object against whom actions are taken to assess their state of consciousness and become increasingly a subject in this assessment. Thus, the character enshrined in the Patient’s Bill of Rights, where they are indicated as a full participant in the treatment/clinical procedure, is fulfilled in such a role.

Although the results of the present research are quite promising, some limitations should be noted. These include the size of the study group. This factor, however, despite the best efforts of the clinical trials, will be considered in future studies. The project’s experience shows that it would be advisable to teach the patient how to use the eye tracker before using it for diagnostic purposes. This will facilitate the patient’s adaptation of their eye muscles to such cooperation, and may subsequently help in a better and more adequate assessment of the patient’s state of consciousness. 

## 5. Conclusions

1. Given the existing significant correlation between the results of the CRS-R test and the results of the MCSD test, it seems reasonable to introduce an assessment of the patient’s state of consciousness based on eye-tracking technology.
2. The applied tool for assessing the patient’s state of consciousness based on eye-tracking technology involves the patient in the procedure of assessing their state of consciousness, making this assessment more dependent than the previously used methods on the cooperation between the patient and the examiner.
3. Incorporating the eye tracker into the neurorehabilitation process may be an important factor in the process of changing the patient’s state of consciousness assessment due to improved control over the muscles used in this assessment. In this approach, the patient becomes the subject of clinical practice to a greater extent than before.
4. The use of modern technology to assess a patient’s state of consciousness opens up the opportunity for greater objectivity, as well as, in the long run, for a reduction in the workload of qualified personnel.

## Figures and Tables

**Table 1:** Basic demographics and clinical data of patients qualified for the MCSD test.

Category		N	%
**Sex**	Men	27	58.7%
	Women	19	41.3%
**Overall**		**46**	**100%**
Patient handedness			
Right-handed		37	80.4%
Left-handed		1	2.2%
No data		8	17.4%
**Overall**		**46**	**100%**
Smoking			
Yes		4	8.7%
No		29	63.0%
No data		13	28.3%
**Overall**		**46**	**100%**
Nature of the brain injury			
Craniocerebral trauma		17	34.0%
Stroke		16	32.0%
Hypoxia due to cardiovascular causes		8	16.0%
Hypoxia due to respiratory causes		5	10.0%
Status epilepticus		2	4.0%
Metabolic disease		1	2.0%
Other		1	2.0%

**Table 2:** Results of GCS and time since injury (months).

	Mean	SD	Min	Q1	Median	Q3	Max
**GCS**	7.0	1.7	3.0	6.0	7.0	8.0	11.0
**Time since injury**	15.7	28.0	0	3.0	6.0	14.0	150.0

**Table 3:** Comparison between the MCSD and the CRS-R results across measurement points, presented as total % scores. T1–T5 indicates the consecutive measurement points.

Time	CRS-R Subscales		Total CRS-R % Score	Total C-EYE X MCSD % Score
			Mean ± SD Median (Q1–Q3)	Mean ± SD Median (Q1–Q3)
**T1**	**T**	10.69 (6.42)	46.47% ± 27.91% 47.83% (21.74–65.22%)	37.17% ± 24.35% 36.36% (18.18–45.45%)
	**Au**	2.0 (1.3)		
	**V**	2.5 (1.8)		
	**M**	2.7 (2.0)		
	**O**	1.2 (1.0)		
	**C**	0.4 (0.6)		
	**A**	1.8 (0.9)		
**T2**	**T**	10.93 (6.06)	47.54% ± 26.36% 43.48% (21.74–65.22%)	34.55% ± 22.90% 27.27% (18.18–36.36%)
	**Au**	2.0 (1.3)		
	**V**	2.5 (1.8)		
	**M**	2.9 (2.0)		
	**O**	1.3 (0.9)		
	**C**	0.3 (0.6)		
	**A**	1.9 (0.8)		
**T3**	**T**	11.00 (6.40)	47.83% ± 27.81% 43.48% (21.74–65.22%)	45.86% ± 20.87% 45.45% (36.36–54.55%)
	**Au**	2.0 (1.3)		
	**V**	2.5 (1.9)		
	**M**	3.0 (2.1)		
	**O**	1.2 (1.0)		
	**C**	0.4 (0.7)		
	**A**	1.9 (0.9)		
**T4**	**T**	11.09 (6.35)	48.21% ± 27.59% 43.48% (21.74–73.91%)	39.19% ± 22.34% 27.27% (27.27–54.55%)
	**Au**	2.1 (1.3)		
	**V**	2.5 (1.8)		
	**M**	2.9 (2.0)		
	**O**	1.3 (0.9)		
	**C**	0.4 (0.7)		
	**A**	1.8 (0.8)		
**T5**	**T**	11.38 (6.57)	49.47% ± 28.58% 56.52% (21.74–69.57%)	41.01% ± 21.81% 36.36% (27.27–54.55%)
	**Au**	2.1 (1.3)		
	**V**	2.6 (1.9)		
	**M**	3.0 (2.0)		
	**O**	1.3 (1.0)		
	**C**	0.4 (0.7)		
	**A**	1.9 (0.9)		
% of patients with DOC diagnosis based on the highest diagnosis across T1-T5 CRS-R administrations: UWS (33.3%), MCS− (24.24%), MCS+ (28.9%), eMCS (13.3%)				
**Relative % change T5 vs. T1**			3.00% ± 10.96% 0.00% (−4.35–4.35%)	3.84% ± 16.95% 0.00% (−9.09–18.18%)
**Sum (T1–T5)** **of Total % points**			47.90% ± 27.04% 47.83% (21.74–66.96%)	39.56% ± 20.35% 34.55% (27.27–45.45%)

Average CRS-R total score and subscale scores at five measurement points (indicated as “Time point” on the table; SD values are given in parentheses). Symbols for CRS-R subscales: T = total result; Au = auditory; V = visual; M = motor; O = oromotor/verbal; C = communication; A = arousal.

**Table 4:** ANOVA-type results of repeated measures comparison between MCSD and CRS-R.

Factor	F Ratio	*p*
**Test type**	2.740	0.1017
**Time**	5.504	0.0007
**Interaction Test × Time**	5.129	0.0012
**Time points comparison**		
**MCSD**	7.061	0.0002
**CRS-R**	1.146	0.3476

**Table 5:** Correlations matrix (Pearson r) for the MCSD and the CRS-R between each time point.

C-Eye X MCSD	T1	T2	T3	T4	T5
**T2**	0.858	–	0.784	0.800	0.739
**T3**	0.724	0.784	–	0.763	0.701
**T4**	0.790	0.800	0.763	–	0.855
**T5**	0.736	0.739	0.701	0.855	–
**CRS-R**	**T1**	**T2**	**T3**	**T4**	**T5**
**T2**	0.976	–	0.945	0.943	0.915
**T3**	0.940	0.945	–	0.960	0.949
**T4**	0.941	0.943	0.960	–	0.964
**T5**	0.925	0.915	0.949	0.964	–

**Table 6:** Results of Passing–Bablok regression between the C-Eye X MCSD and the CRS-R at each time point and for the sum from T1 to T5.

Time Point	β_0_	±95% CI for β_0_	β_1_	±95% CI for β_1_
**T1**	1.60	−14.58–19.48	0.73	0.35–1.04
**T2**	3.03	−9.09–9.92	0.70	0.42–1.04
**T3**	12.12	3.63–26.26	0.70	0.44–0.92
**T4**	1.14	−12.12–15.91	0.78	0.52–1.04
**T5**	9.09	−2.27–17.53	0.70	0.42–0.93
**Sum T1–T5**	12.98	−0.64–20.83	0.57	0.33–0.85

β_0_—regression coefficient—intercept; β_1_—regression coefficient—slope; CI—confidence interval.

## Data Availability

Data and methods of analysis are available to qualified researchers upon request.
